# Structure of the *Zymomonas mobilis* respiratory chain: oxygen affinity of electron transport and the role of cytochrome *c* peroxidase

**DOI:** 10.1099/mic.0.081612-0

**Published:** 2014-09

**Authors:** Elina Balodite, Inese Strazdina, Nina Galinina, Samantha McLean, Reinis Rutkis, Robert K. Poole, Uldis Kalnenieks

**Affiliations:** 1 Institute of Microbiology and Biotechnology, University of Latvia, Kronvalda boulv. 4, 1586 Riga, Latvia; 2 Department of Molecular Biology and Biotechnology, University of Sheffield, Firth Court, Western Bank, Sheffield S10 2TN, UK

## Abstract

The genome of the ethanol-producing bacterium *Zymomonas mobilis* encodes a *bd*-type terminal oxidase, cytochrome *bc*_1_ complex and several *c*-type cytochromes, yet lacks sequences homologous to any of the known bacterial cytochrome *c* oxidase genes. Recently, it was suggested that a putative respiratory cytochrome *c* peroxidase, receiving electrons from the cytochrome *bc*_1_ complex via cytochrome *c*_552_, might function as a peroxidase and/or an alternative oxidase. The present study was designed to test this hypothesis, by construction of a cytochrome *c* peroxidase mutant (Zm6-*perC*), and comparison of its properties with those of a mutant defective in the cytochrome *b* subunit of the *bc*_1_ complex (Zm6-*cytB*). Disruption of the cytochrome *c* peroxidase gene (ZZ60192) caused a decrease of the membrane NADH peroxidase activity, impaired the resistance of growing culture to exogenous hydrogen peroxide and hampered aerobic growth. However, this mutation did not affect the activity or oxygen affinity of the respiratory chain, or the kinetics of cytochrome *d* reduction. Furthermore, the peroxide resistance and membrane NADH peroxidase activity of strain Zm6-*cytB* had not decreased, but both the oxygen affinity of electron transport and the kinetics of cytochrome *d* reduction were affected. It is therefore concluded that the cytochrome *c* peroxidase does not terminate the cytochrome *bc*_1_ branch of *Z. mobilis*, and that it is functioning as a quinol peroxidase.

## Introduction

Over the last few decades, the ethanol-producing bacterium *Zymomonas mobilis* has been an object of ongoing interest in biotechnology (Swings & deLey, 1977; Rogers *et al.*, 1982). Recently, full genome sequences of several *Z. mobilis* strains have become available (Seo *et al.*, 2005; Kouvelis *et al.*, 2009; Pappas *et al.*, 2011; Desiniotis *et al.*, 2012). The high specific rates of sugar uptake and ethanol fermentation by *Z. mobilis*, and its relatively small genome size, make it a promising candidate for metabolic engineering of pathways for bioethanol synthesis from agricultural and forestry waste (Dien *et al.*, 2003; Rogers *et al.*, 2007; Lau *et al.*, 2010). However, for wider applications in novel bioprocesses, a more in-depth understanding of its energy metabolism would be needed, in particular concerning its aerobic metabolism.

This bacterium possesses a constitutive electron transport chain with a relatively high rate of oxygen consumption, but a low apparent yield of ATP (Bringer *et al.*, 1984; Kalnenieks *et al.*, 1993). Neither the physiological function of the respiratory chain nor the mechanistic reasons for the low coupling efficiency of oxidative phosphorylation in *Z. mobilis* have been sufficiently elucidated (Kalnenieks, 2006). In part, this is because the organization of respiratory components and the routes for electron transfer to oxygen remain unresolved. Based on genomic information, there is only one functional respiratory NAD(P)H dehydrogenase in the *Z. mobilis* electron transport chain, belonging to the type II respiratory dehydrogenase (Ndh) family (Kalnenieks *et al.*, 2008; Yang *et al.*, 2009), and only one terminal cytochrome *bd*-type quinol oxidase has been identified so far (Kalnenieks *et al.*, 1998; Seo *et al.*, 2005; Sootsuwan *et al.*, 2008). The known *Z. mobilis* genome sequences also contain genes encoding a cytochrome *bc*_1_ complex and several genes for *c*-type cytochromes, yet lack sequences homologous to any known bacterial cytochrome *c* oxidase genes.

Recently, mutants of the cytochrome *bc*_1_ complex and of the *bd* terminal oxidase were constructed and studied (Strazdina *et al.*, 2012). Both mutants showed strongly altered respiratory phenotypes. With two functional branches of electron transport chain, the lack of genes for terminal oxidases other than cytochrome *bd* raises the intriguing problem of what could be the nature of the oxidase terminating the cytochrome *bc*_1_ branch. Sootsuwan *et al.* (2008) and Charoensuk *et al.* (2011) proposed that the cytochrome *bc*_1_ branch is probably terminated by a cytochrome *c* peroxidase (PerC). The corresponding candidate gene is present in the genome (ZZ60192). We speculated that the cytochrome *c* peroxidase gene product might indeed substitute for a ‘proper’ alternative oxidase (Strazdina *et al.*, 2012), which in principle could happen in different ways. First, it might have side-activity of an oxidase, although that would be most unusual and, to the best of our knowledge, has never been reported for a cytochrome *c* peroxidase. Second, it could function in combination with a respiratory peroxide-generating reaction, like the one reported for *Escherichia coli* fumarate reductase under aerobic conditions (Korshunov & Imlay, 2010). We aimed to test these hypotheses to establish the relevance of PerC to the alternative oxidase activity and the cytochrome *bc*_1_ branch in *Z. mobilis*. For that, construction of a ZZ60192 null mutant was necessary. In the present work, we report construction and study of such a cytochrome *c* peroxidase mutation in the *Z. mobilis* centrotype strain ATCC 29191. We set out to: (i) establish the presence of the product of gene ZZ60192 (*perC*) in membranes, using cytochrome redox differential spectroscopy; (ii) verify the putative role of perC in the electron transport to H_2_O_2_ and/or oxygen, and in the protection of cells against exogenous hydrogen peroxide (H_2_O_2_); and (iii) determine whether the cytochrome *bc*_1_ complex serves as the electron donor for the cytochrome *c* peroxidase, by comparing the respiratory parameters of the respective mutants.

## Methods

### 

#### Bacterial strains, plasmids, and transformation.

*E. coli* JM109 and plasmid pGEM-3Zf(+) were purchased from Promega. Strain JM109 was used as the host for cloning of the recombinant plasmids. *Z. mobilis* ATCC 29191 (Zm6) and its mutant derivative, defective in the cytochrome *b* subunit of the *bc*_1_ complex (strain Zm6-*cytB*), were maintained and cultivated as described previously (Kalnenieks *et al.*, 1993; Strazdina *et al.*, 2012). The plasmids and *Z. mobilis* strains constructed and used in the present work are listed in Table 1. *E. coli* was transformed by the CaCl_2_ procedure described by Sambrook *et al.* (1989). *Z. mobilis* was transformed by electroporation (Liang & Lee, 1998).

**Table 1.** Plasmids and strains used in the study

Plasmid/strain	Characteristics	Source
pGEM-3Zf(+)	Amp^r^	Promega
pBT	Cm^r^	Stratagene
pGEMperC	pGEM-3Zf(+) derivative, carrying a 1.34 kb fragment of PCR-amplified genomic DNA with the ORF of the cytochrome *c* peroxidase gene (*perC*; ZZ60192) cloned between the *Bam*HI and *Hin*dIII sites of the MCS	Present work
pGEMperC : : cm^r^	pGEMperC derivative, carrying in the *Age*I site of the *perC* a 1.3 kb *Age*I restriction fragment of pBT, with a 0.7 kb chloramphenicol-resistance ORF	Present work
pBBR1MCS-2	Kan^r^	NCBI GenBank
		U23751
pBBRperC	pBBR1MCS-2 derivative, carrying a 1.57 kb fragment of PCR-amplified genomic DNA with the cytochrome *c* peroxidase gene (*perC*; ZZ60192) and its promoter region, cloned between *Hin*dIII and *Bam*HI sites of the MCS	Present work
Zm6	Parent strain	ATCC 29191
Zm6-*cytB*	Zm6 strain with a Cm^r^ insert in the ORF of the cytochrome *b* subunit gene (ZMO 0957) of the *bc*_1_ complex	Strazdina *et al.* (2012)
Zm6-*perC*	Zm6 strain with a Cm^r^ insert in the ORF of *perC*	Present work
Zm6-*perC*pBBRperC	Zm6-*perC*, carrying plasmid pBBRperC	Present work

#### Cloning techniques, PCR and mutant construction.

Genomic and plasmid DNA isolation from *Z. mobilis* were performed as before (Kalnenieks *et al.*, 2008; Strazdina *et al.*, 2012). The *Z. mobilis* putative cytochrome *c* peroxidase gene (*perC*; *Z. mobilis* Zm6 genome sequence, locus tag ZZ60192) was amplified by PCR using the primer pair cytperox1 (CTTCTTCTGGGATCCTTGCCAGATTATGGC) and cytperox2 (GCCTATGGGGCAACAAGCTTTTATCTGGTTC). The engineered restriction sites for *Bam*HI and *Hin*dIII, respectively, are underlined. To obtain a mutant defective in the *perC* gene (Zm6-*perC*), the amplified 1.34 kb region of the chromosomal DNA containing the *perC* ORF was double-digested with *Bam*HI and *Hin*dIII, and directionally cloned between the *Bam*HI and *Hin*dIII restriction sites of the multiple cloning site (MCS) of plasmid pGEM-3Zf(+), yielding plasmid pGEMperC (Table 1). The plasmid was used to transform *E. coli* JM109, and the transformants were plated on Luria–Bertani agar with ampicillin (100 μg ml^−1^). Plasmid pBT (Table 1) was digested with *Age*I, and the 1.27 kb restriction fragment, containing a 659 bp ORF of the chloramphenicol acetyltransferase gene, was inserted into the *Age*I site of the cloned *perC* gene, yielding plasmid pGEMperC : : cm^r^ (Table 1). This plasmid, unable to propagate in *Z. mobilis*, was used to transform *Z. mobilis* by electroporation, and homologous recombinants were selected on plates containing chloramphenicol (120 µg ml^−1^).

#### Verification and complementation of the mutant strain.

After transformation, several colonies growing on plates with chloramphenicol were screened for the *perC* : : cm^r^ genotype by PCR on the genomic DNA template with primers cytperox1 and cytperox2, yielding an amplified DNA fragment an extra 1.3 kb in length. Insertion of the chloramphenicol-resistance determinant in the cytochrome *c* peroxidase gene was further verified by sequencing the PCR product. For complementation of the knockout mutant Zm6-*perC*, a 1.57 kb chromosomal DNA fragment, containing *perC* with its promoter region, was amplified, using the primer pair ZZ60192f (TGTTAAGCTTCAATAAATAAAAAGGT) and ZZ60192r (TTAAAGAGGATCCTGATTATTTAGAA). The engineered restriction sites for *Hin*dIII and *Bam*HI, respectively, are underlined. The amplified fragment was double-digested with *Bam*HI and *Hin*dIII, and directionally cloned between the *Hin*dIII and *Bam*HI restriction sites of the MCS of shuttle vector pBBR1MCS-2 yielding plasmid pBBRperC (Table 1). Plasmid pBBRperC was used for transformation of Zm6-*perC* by electroporation, and transformants were selected on agar plates containing chloramphenicol (120 µg ml^−1^) and kanamycin (310 µg ml^−1^). Total DNA of the transformed strains was isolated, and the presence of intact ZZ60192 was verified by PCR with the primer pair cytperox1 and cytperox2.

Primers for PCRs were supplied by Operon and Invitrogen. PCRs were carried out in an Eppendorf Mastercycler, using Fermentas *Taq* DNA polymerase. Other DNA manipulations were carried out as described previously (Kalnenieks *et al.*, 2008; Strazdina *et al.*, 2012), using Qiagen kits. All DNA constructs were confirmed by DNA sequencing, carried out by Beckman Coulter genomics.

#### Cultivation, preparation of membranes and cytochrome spectroscopy.

The growth medium contained glucose (20 g l^−1^), yeast extract (5 g l^−1^) and mineral salts, as described previously (Kalnenieks *et al.*, 1993). Cultivations were carried out on a shaker at 100 r.p.m. in 200 ml shaken flasks, containing 30 ml of culture. Membranes were prepared by ultrasonic breakage of cells, followed by centrifugation steps, as described previously (Strazdina *et al.*, 2012). Room temperature reduced minus oxidized (‘as prepared’) cytochrome absorption spectra were taken using membrane samples (2.5 ml) at a protein concentration of 5–6 mg ml^−1^, adding 25 µl of 0.5 M NADH as the reductant. Spectra were recorded with an Olis RSM1000 dual-beam rapid scanning monochromator (Online Instrument Systems), which permits the rapid acquisition of up to 1000 absorbance scans per second over a wavelength range of 300 nm, giving extremely fast time-resolved spectra or it allows the generation of average scans from the many taken over a period of time, greatly increasing the signal-to-noise ratio. The time course of cytochrome *d* reduction after addition of NADH was recorded by rapid, repetitive scanning in the wavelength range between 400 and 700 nm every 10 s, acquiring 1000 scans s^−1^ (averaged to give one scan per time point). The degree of cytochrome *d* reduction was calculated as the mean value of the absorbance differences at wavelength pairs 630/614 and 630/646 nm, while wavelength pairs 550/545 and 560/575 nm were used to calculate the overall degree of *c*- and *b*-type cytochrome reduction.

#### Determination of oxygen affinities.

**Fig. 1.**
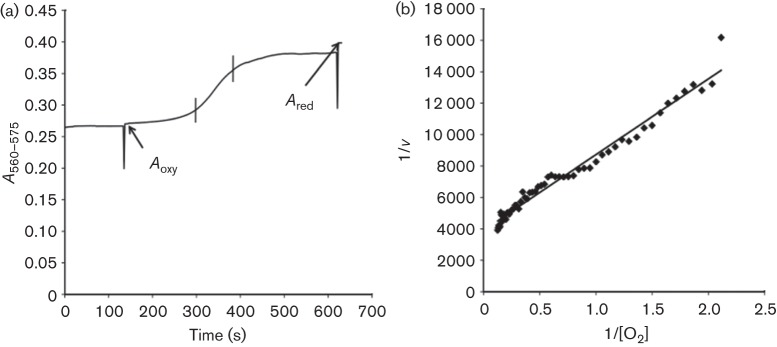
Kinetics of myoglobin deoxygenation resulting from oxygen consumption by cytoplasmic membrane preparation. (a) A typical time course of the absorbance difference at 575/560 nm with Zm6 membranes; *A*_oxy_ is the absorbance difference recorded immediately after addition of membranes into the assay with oxygenated myoglobin; *A*_red_ is the absorbance difference reached after deoxygenation of myoglobin and addition of a small amount of reductant (dithionite). (b) The absorbance differences [between the two vertical bars in (a)] were taken for calculations and for building of Lineweaver–Burk plots of oxygen consumption rate versus the free oxygen concentration, used to find the *K*_m_ values (here the calculated *K*_m_ value is 1.24 µM).

Oxygen affinity of the *Z. mobilis* respiratory chain was determined by monitoring the deoxygenation kinetics of sperm-whale oxymyoglobin, essentially following the routine described by D’Mello *et al.* (1994). A custom-made, sealed optical cuvette (1.3 ml total volume, 1 cm light pathlength) was filled with 100 mM potassium phosphate buffer, previously deoxygenated by gassing with nitrogen. A few microlitres of NADH and oxymyoglobin stock solutions were added to the cuvette via a small hole in the lid using a Hamilton syringe, to yield final concentrations of 2 mM and 10 µM, respectively, and the cuvette was placed on a magnetic stirrer in the SDB-4 dual-wavelength spectrophotometer. Stability of the oxygenated form of myoglobin was checked by recording the absorbance difference between 575 and 560 nm for several minutes (Bergersen & Turner, 1979; Wood, 1984). After addition of membranes (10–100 µl), the 575/560 nm absorbance difference was further monitored, to follow deoxygenation kinetics of the oxygenated myoglobin (Fig. 1a). At least five separate determinations were carried out on each membrane preparation. The rate of oxygen consumption and the concentration of free dissolved oxygen at each time point were calculated according to Bergersen & Turner (1979) using Microsoft Excel software. In brief, the fractional oxygenation of myoglobin at any moment of time, *Y*_t_ = [MbO_2_]/([MbO_2_]+[Mb]), can be estimated from the measured absorbance ratio (*A*_t_−*A*_red_)/(*A*_oxy_−A_red_), where *A*_t_ is the 575/560 nm absorbance difference at the respective time point, *A*_oxy_ is the absorbance difference with all myoglobin being in the oxygenated state, and *A*_red_ is the same with all myoglobin being deoxygenated (Fig. 1a). Taking 0.786 µM for the oxymyoglobin dissociation constant, *K* = ([Mb][O_2_])/[MbO_2_] (Bergersen & Turner 1979), the explicit relationship between the concentration of free oxygen and the fractional oxygenation of myoglobin would be: [O_2_] = 0.786*Y*_t_/(1−*Y*_t_). The total amount of oxygen can be calculated from the equation: O_2 total_ = *V*(*Y*_t_*C*+[O_2_]), where *V* is the volume of the cuvette (1.3 ml in our case) and *C* is the total concentration of myoglobin, estimated from the 435/420 nm absorbance difference in the CO difference spectrum of the dithionite-reduced myoglobin sample (Wood, 1984).

To find the apparent *K*_m_ for oxygen, the calculated values of free dissolved oxygen concentration were plotted in Lineweaver–Burk coordinates versus the corresponding rates of decrease of the total oxygen amount in the cuvette (Fig. 1b). The values of *A*_t_ for the calculations were taken from the time interval in which the most rapid change of this parameter occurred (between the two vertical bars in Fig. 1a). Such *K*_m_ values represent average estimates for the entire respiratory chain. The exact number and the nature of terminal oxidases in *Z. mobilis* remain uncertain, and therefore we did not attempt to identify the contributions of putative individual terminal oxidases by using Eadie–Hofstee plots.

#### Analytical methods.

H_2_O_2_ production by cells was determined fluorimetrically by monitoring Amplex UltraRed fluorescence during its reaction with H_2_O_2_, catalysed by horseradish peroxidase (Korshunov & Imlay, 2010). Fluorescence was measured with a FluoroMax-3 spectrofluorimeter (Jobin–Yvon), using 520 nm wavelength for excitation and 620 nm wavelength for emission. To quantify the generated H_2_O_2_, fluorescence increase was calibrated by addition of 1 mM H_2_O_2_ in 5 µl increments. The NADH oxidase assay for membranes was carried out by monitoring NADH oxidation spectrophotometrically at 340 nm, as previously described (Kalnenieks *et al.*, 2008). Measurement of the NADH peroxidase activity was done generally following the assay described by Yamada *et al.* (2007). After deoxygenation by gassing the cuvette with oxygen-free nitrogen, the peroxidase reaction was started by addition of 10 mM H_2_O_2_ (to a final concentration of 0.01 mM) to the assay mixture containing the membranes, 1 mM glucose and 50 units of glucose oxidase, and NADH oxidation was monitored at 340 nm. For whole-cell oxygen consumption measurements, the concentration of dissolved oxygen was monitored using a Radiometer Clark-type oxygen electrode. Protein concentration in cell-free extracts and membrane samples was determined according to Markwell *et al.* (1978). Cell concentration was determined as OD_550_, and dry cell mass of the suspensions was calculated by reference to a calibration curve. Results were means of at least three replicates. Oxygen affinity measurements were done in five to eight replicates. Error bars in the figures represent sem.

## Results

### H_2_O_2_ sensitivity

**Fig. 2.**
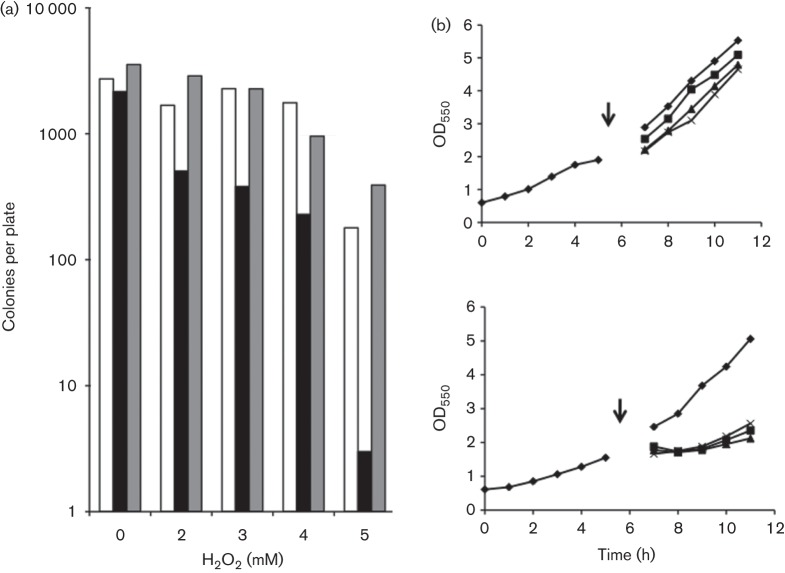
Effect of various concentrations of H_2_O_2_ on aerobic growth and survival. (a) Colony counts in H_2_O_2_ killing assay: open bars, Zm6; black bars, Zm6-*perC*; grey bars, Zm6-*perC*pBBRperC. (b) Aerobic growth of batch cultures of Zm6 (top) and Zm6-*perC* (bottom) after transfer into fresh growth media following H_2_O_2_ addition (denoted by arrows) at (⧫) 0 mM, (□) 0.5 mM, (▴) 1.0 mM and (×) 1.5 mM final concentration.

An H_2_O_2_ killing assay was carried out with cultures grown in shaken flasks at 200 r.p.m. to an OD_550_ of 2, corresponding to late exponential phase. Cultures were split into aliquots and transferred to Eppendorf tubes, and H_2_O_2_ was added to final concentrations as indicated in Fig. 2(a). After incubation for 30 min at 30 °C, suspensions were diluted 400 times, 6 µl aliquots were spread on agar plates with appropriate antibiotics and colonies were counted after incubation overnight. As seen from Fig. 2(a), the sensitivity of Zm6-*perC* to H_2_O_2_ had significantly increased. At 5 mM H_2_O_2_ in the medium the survival rate of the mutant was below 2 % of that of Zm6. However, complementation of the mutation fully restored the initial resistance to H_2_O_2_ killing.

In the peroxide sensitivity assay, shown in Fig. 2(b), growing cells were harvested by centrifugation and resuspended in fresh growth medium supplemented with various concentrations of H_2_O_2_ in the millimolar range. Also in this assay, sensitivity of growing Zm6-*perC* to externally added H_2_O_2_ appeared to be higher than that seen with Zm6 and surpassed even that of the previously constructed catalase-deficient strain (Strazdina *et al.*, 2012). Its growth was stopped even at 0.5 mM H_2_O_2_, although catalase activity in Zm6-*perC* was the same as in Zm6 (data not shown). Note that, in contrast to Zm6-*perC*, the H_2_O_2_ sensitivity of growing Zm6-*cytB* culture, using the same sensitivity assay, was previously found to be similar to that of the parent strain (Strazdina *et al.*, 2012).

### Function of cytochrome *c* peroxidase in the respiratory chain

As the ZZ60192 product showed a distinct physiological effect on H_2_O_2_ sensitivity, our next step was to relate its function to the respiratory chain. Previously it was found that in *Z. mobilis* cytochrome *c* peroxidase could not be detected by the standard assay based on H_2_O_2_-dependent oxidation of externally added cytochrome *c* in cell-free extracts (Charoensuk *et al.*, 2011; Strazdina *et al.*, 2012). We therefore tried to establish whether the null mutation had any effect on the cytochrome content and function of the respiratory chain.

The mutation did not alter the kinetic parameters of oxygen consumption by cytoplasmic membrane preparations. Both Zm6 and Zm6-*perC* showed NADH oxidase activity very close to 0.5 U per milligram of membrane protein (Table 2). Fig. 1(a) shows a typical time course of 575/560 nm absorbance difference during deoxygenation of myoglobin in the presence of Zm6 membrane preparation, and Fig. 1(b) shows the corresponding Lineweaver–Burk graph used for calculation of the apparent *K*_m_ for oxygen. The apparent *K*_m_ values for oxygen in membrane preparations of strains Zm6 and Zm6-*perC* did not differ significantly, being around 1.2 µM (Table 2). Notably, in Zm6-*cytB*, the strain with the disrupted gene for the cytochrome *b* subunit of the *bc*_1_ complex, the NADH oxidase activity was in the same range, while the *K*_m_ for oxygen was substantially lower, close to 0.4 µM. The difference between the mean *K*_m_ values of Zm6 and Zm6-*cytB*, as well as between those of Zm6-*perC* and Zm6-*cytB*, was statistically significant (*P*<0.01 for both cases).

**Table 2.** Kinetics of NADH oxidation in *Z. mobilis* membrane preparations with H_2_O_2_ and O_2_ as electron acceptors Values are shown as mean (±sem).

Strain	NADH oxidation [U (mg protein)^−1^]		*K*_m_ (µM O_2_)
	NADH peroxidase	NADH oxidase	
Zm6	0.114 (±0.008)	0.491 (±0.092)	1.28 (±0.242)
Zm6-*perC*	0.072 (±0.010)	0.499 (±0.023)	1.18 (±0.365)
Zm6-*cytB*	0.128 (±0.007)	0.543 (±0.007)	0.38 (±0.079)

**Fig. 3.**
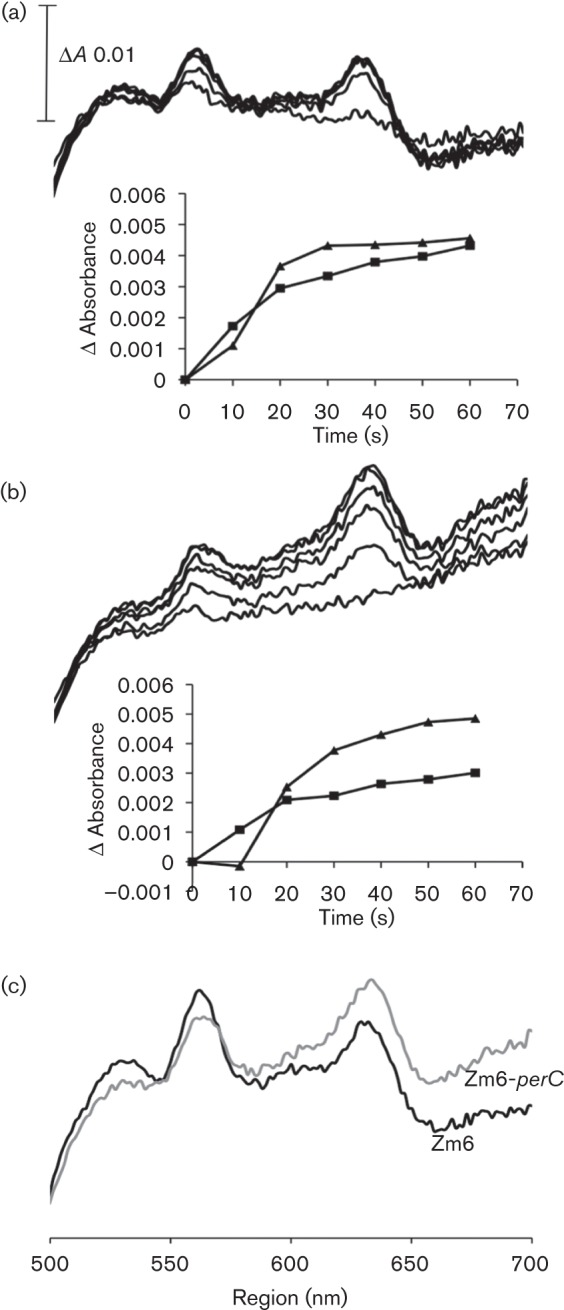
Cytochrome reduction with NADH in membrane preparations. (a) Time course of cytochrome reduction in membranes of Zm6, and (b) the same in membranes of Zm6-*perC*; spectra were recorded at 10 s intervals during the first minute after NADH addition. Insets: the time course of absorbance of cytochrome *d* (mean absorbance differences at wavelength pairs 630/614 and 630/646 nm) (▴), and of *c*- and *b*-type cytochromes (the wavelength pairs 560/545 and 560/575 nm) (□). (c) The cytochrome spectra 3 min after NADH addition.

 ‘As prepared’ visible-light absorption spectra of membrane preparations of the parent strain and the mutant were recorded, using NADH as the reductant (Fig. 3). The cytochrome *c* peroxidase deficiency clearly manifested itself in the difference spectra of the mutant membranes: in the alpha region between 545 and 560 nm, corresponding largely to *c*-type cytochromes, absorbance was decreased. For Zm6, absorbance in this spectral region 1 min after NADH addition reached a higher level (Fig. 3a) than for Zm6-*perC* (Fig. 3b), and accordingly the stationary cytochrome reduction levels attained after 3 min markedly differed between the two strains (Fig. 3c). At the same time, *perC* disruption had much less effect on cytochrome *d* reduction. The cytochrome *d* spectral signal around 630 nm 1 min after NADH addition, as well as the final absorbance values reached after 3 min, was similar in both strains.

### NADH-dependent membrane peroxidase activity

Membrane preparations of Zm6 in a cuvette, flushed with nitrogen gas and supplemented with excessive amounts of glucose and glucose oxidase for oxygen removal, were able to oxidize NADH in response to H_2_O_2_ addition (Table 2). Similar observations were reported by Charoensuk *et al.* (2011) for a thermotolerant *Z. mobilis* strain. Zm6-*perC* membranes in such an assay showed a significantly lower (*P*<0.05) rate of NADH oxidation than that of the parent strain. Nevertheless, the *perC* disruption did not eliminate all of the apparent H_2_O_2_-dependent NADH-oxidizing activity. We speculate that at least part of the remaining activity in the mutant membranes could be due to the NADH oxidase activity of the electron transport chain, which might be supplied with trace amounts of oxygen coming from slow decomposition of H_2_O_2_. The presence of oxygen in the assay at very low concentration seems plausible also because of low oxygen affinity of the oxygen-removing enzyme: the *K*_m_ of glucose oxidase for oxygen has been reported to be in the several hundred micromolar range (Nakamura *et al.*, 1976), while for the electron transport chain, according to our data, *K*_m_ is around 1 µM. In comparison to strain Zm6, however, the NADH peroxidase assay in strain Zm6-*cytB* indicated no decrease, but rather an increase of activity (Table 2). Accordingly, the difference between strains Zm6-*perC* and Zm6-*cytB* was even more marked (*P*<0.01).

### Aerobic growth and respiration

**Fig. 4.**
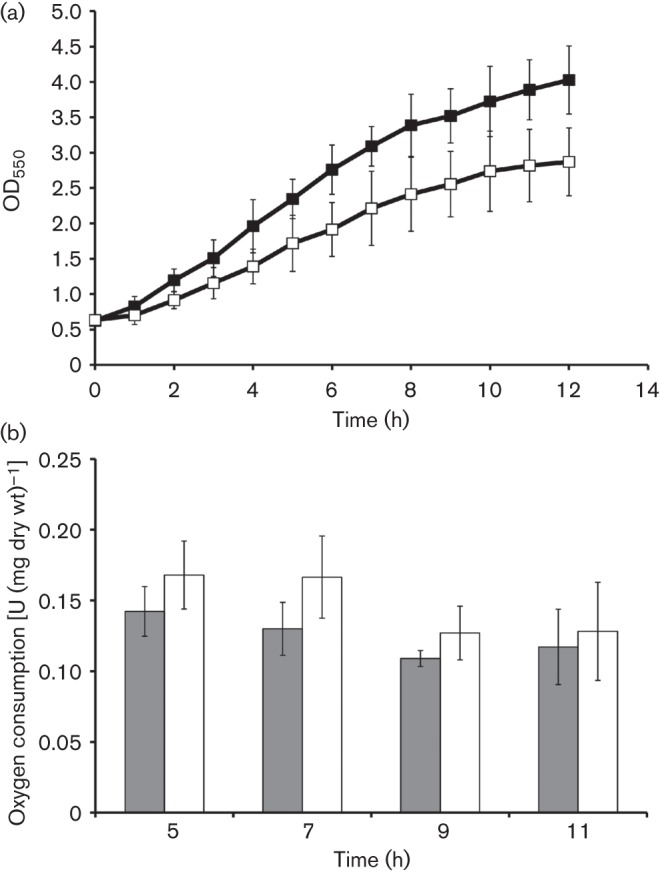
Aerobic growth and oxygen consumption. (a) Aerobic batch cultivation of Zm6 (▪) and Zm6-*perC* (□). (b) Oxygen consumption in washed cell suspension of Zm6 (filled bars) and Zm6-*perC* (empty bars) with ethanol (10 g l^−1^).

Aerobic growth and respiratory capacity of whole cells was monitored during batch cultivations (Fig. 4). For the respiration assay, 1.5 ml samples were taken after 5, 7, 9 and 11 h of cultivation. Cells were sedimented, washed and resuspended in 100 mM phosphate buffer, pH 7, and oxygen consumption was measured with ethanol (10 g l^−1^) as electron donor. Mutating *perC* did not cause any loss of respiratory activity. Instead, a slight increase of respiration rate was noted in Zm6-*perC*. Likewise, mutation had no effect on H_2_O_2_ evolution from cells. Cells of both strains, when suspended in phosphate buffer with 20 g glucose l^−1^, excreted H_2_O_2_ at a low rate, close to 0.02 nmol min^−1^ (mg dry wt)^−1^ (data not shown). The mutant strain, nevertheless, grew more slowly and reached a lower biomass concentration at the end of batch growth (Fig. 4).

## Discussion

Our results support the presence of a functional cytochrome *c* peroxidase, the product of *perC* (ZZ60192), in the membranes of *Z. mobilis* Zm6 (ATTC 29191). Cytochrome *c* peroxidase deficiency in the mutant strain Zm6-*perC* was detectable in the difference spectra of membranes, and manifested itself via a partial loss of the membrane NADH peroxidase activity, a decrease of aerobic growth rate and an increased sensitivity of cells to externally added H_2_O_2_. Elevated H_2_O_2_ sensitivity, severe disturbance of aerobic growth at high temperature and loss of respiratory peroxidase activity were reported by Charoensuk *et al.* (2011) for a thermotolerant *Z. mobilis perC* mutant strain. Cytochrome *c* peroxidase is thus physiologically important for *Z. mobilis* oxidative stress tolerance, and its deficiency is poorly compensated for by alternative stress-protection systems. However, the present work was intended primarily to check *Z. mobilis* for a novel, non-standard role of a bacterial cytochrome *c* peroxidase. We were interested in whether PerC could serve as a substitute for cytochrome *c* oxidase function in the respiratory chain of this bacterium.

Here we demonstrated that the *perC* mutation had no significant effect upon: (i) the kinetics of cytochrome *d* reduction, (ii) the apparent *K*_m_ for oxygen in membrane preparations, (iii) the respiratory capacity of growing cells and (iv) the excretion of H_2_O_2_ by the cells. Together these findings indicate that the product of the *perC* (ZZ60192) gene is not participating in electron transfer to oxygen – neither directly, nor in combination with a putative respiratory H_2_O_2_-generating reaction. Hence, *perC* seems not to be the solution for the puzzle of the ‘hidden’ alternative oxidase of *Z. mobilis*. At the same time, the *cytB* mutation did affect the apparent *K*_m_ value for oxygen and, as reported by Strazdina *et al.* (2012), also the redox state of cytochrome *d*. In contrast to *perC*, the *cytB* mutation did not cause any loss of membrane NADH peroxidase activity and also, as previously shown (Strazdina *et al.*, 2012), had no effect on the H_2_O_2_ sensitivity of cells. Therefore, it seems apparent that: (i) an unidentified oxidase, but not cytochrome *c* peroxidase, is terminating the cytochrome *bc*_1_ branch, and (ii) the cytochrome *bc*_1_ complex is not the major supplier of electrons for the *Z. mobilis* cytochrome *c* peroxidase.

The cytochrome *c* peroxidase of *Z. mobilis* belongs to the family of bacterial peroxidases with three haem-binding motifs. These cytochrome *c* peroxidases carry an N-terminal extension with a third haem *c*-binding motif (CXXCH) and a methionine ligand (Atack & Kelly, 2006). The tri-haem cytochrome *c* peroxidase in *Aggregatibacter actinomycetemcomitans* has been shown to be a quinol peroxidase (Yamada *et al.*, 2007; Takashima & Konishi, 2008), and we assume the same also for *Z. mobilis*. Yet, based on inhibitor analysis with antimycin, Charoensuk *et al.* (2011) concluded that for *Z. mobilis* enzyme the cytochrome *bc*_1_ complex is the major source of electrons, with NADH as the reductant. They found that the H_2_O_2_-dependent oxidation of NADH in the membranes was highly sensitive to 50 µM antimycin, while the more rapid H_2_O_2_-dependent oxidation of externally added quinol was less sensitive to this inhibitor. It is known, however, that antimycin acts as a competitive inhibitor also for quinone-binding sites of several other bacterial electron transport components apart from the cytochrome *bc*_1_ complex. For example, 50 µM antimycin inhibits *E. coli* cytochrome *bd* terminal oxidase by 80 % and cytochrome *bo* terminal oxidase by 18 % (Meunier *et al.*, 1995), even though this bacterium lacks a cytochrome *bc*_1_ complex. Accordingly, binding of antimycin directly to the putative quinol-binding site of the cytochrome *c* peroxidase would be a reasonable alternative explanation for the data of Charoensuk *et al.* (2011)*.* In that case, at high externally added quinol concentrations competitive inhibition should be less pronounced, while with NADH, presumably generating lower quinol concentration, the inhibitory effect of antimycin should be more marked, as was indeed observed. Hence, quinol is a more likely supplier of electrons to PerC than the *bc*_1_ complex, and so the pathway of electrons from the cytochrome *bc*_1_ complex to oxygen in this bacterium remains to be elucidated.
